# Evaluation of the Antimicrobial Protection of Pharmaceutical Kaolin and Talc Modified with Copper and Zinc

**DOI:** 10.3390/ma14051173

**Published:** 2021-03-02

**Authors:** Fotini Martsouka, Konstantinos Papagiannopoulos, Sophia Hatziantoniou, Martin Barlog, Giorgos Lagiopoulos, Athanasia G. Tekerlekopoulou, Dimitrios Papoulis

**Affiliations:** 1 Department of Geology, University of Patras, 26504 Patras, Greece; geo13118@upnet.gr (K.P.); papoulis@upatras.gr (D.P.); 2 Department of Pharmacy, University of Patras, 26504 Patras, Greece; sohatzi@upatras.gr; 3 Institute of Inorganic Chemistry, Slovak Academy of Sciences (SAS), Dúbravská cesta 9, 845 36 Bratislava, Slovakia; martin.barlog@savba.sk; 4 Microbiology Department, Quality Assurance and Control Systems—QACS Labs, Antigonis 1, 14451 Metamorfosis, Greece; geolag@qacs.gr; 5 Department of Environmental Engineering, University of Patras, 2 G. Seferi Str, 30100 Agrinio, Greece; atekerle@upatras.gr

**Keywords:** antimicrobial protection, copper, kaolin, talc, zinc

## Abstract

Six pharmaceutical pastes were prepared using chemically modified kaolin and talc powders. Tests were conducted to determine their structural and chemical characteristics as well as their antimicrobial protection, thus rendering them suitable for cosmetic and pharmaceutical uses. Kaolin and talc were treated chemically via the cation exchange method to load the clay particles with copper and zinc ions, two cations well known for their antimicrobial properties. Mineralogical analyses were conducted by using X-ray diffraction (XRD) before and after the modification, confirming the mineralogical purity of the samples. Scanning electron microscopy was also used in conjunction with energy dispersed spectroscopy (SEM-EDS) to obtain chemical mapping images, revealing the dispersion of the added metals upon the clay minerals surfaces. Moreover, chemical analysis has been performed (XRF) to validate the enrichment of the clays with each metal utilizing the cation exchange capacity. All modified samples showed the expected elevated concentration in copper or zinc in comparison to their unmodified versions. From the X-ray photoelectron spectroscopy (XPS), the chemical state of the samples’ surfaces was investigated, revealing the presence of salt compounds and indicating the oxidation state of adsorbed metals. Finally, the resistance of pastes in microbial growth when challenged with bacteria, molds, and yeasts was assessed. The evaluation is based on the European Pharmacopeia (EP) criteria.

## 1. Introduction

Clay minerals exhibit interest in the pharmaceutical industry mainly due to their physicochemical properties [1,2,3]. Since ancient times, they are well-known for their pharmaceutical and cosmetic applications [4,5,6]. These are used either as active ingredients (showing healing properties) or as excipients (accompanying auxiliary substances) in various pharmaceutical and cosmetic preparations [3].

Kaolinite and talc are the main two that have been reported by various authors [7,8,9,10,11,12,13,14,15,16]. Kaolinite is a 1:1 phyllosilicate clay mineral, with the chemical composition Al_2_Si_2_O_5_(OH)_4_. It is a soft mineral with a hardness of 2–2.5 on the Mohs scale, is opaque, mainly white to gray color, and sometimes red, blue, or brown tints from impurities, with particle sizes of 0.2–12 μm, cation exchange capacity of 3–15 mEq/100 g [17,18,19,20,21,22], and specific surface area of about 5–20 m^2^ g^−1^ [18,19,20,21,22,23,24]. Kaolinite’s characteristic property is the ability to absorb proteins, bacteria, and even viruses on the surfaces of its crystals, which can be easily removed [25,26,27,28,29,30]. Talc is a 2:1 phyllosilicate clay mineral, with the chemical composition Mg_3_Si_4_O_10_(OH)_2_. It is the softest mineral with a hardness of 1 on the Mohs scale. It is often also found in pharmaceutical and cosmetic products. The pharmaceutical grade talc is white to light gray opaque, with a small particle size, cation exchange capacity of 0–2 mEq/100 g [18,19,20,21,22], and medium specific surface area 1–10 m^2^ g^−1^ [18,19,20,21,22]. These physical and physicochemical properties of kaolinite and talc render them suitable for their use in pharmaceutical and cosmetic products for topical administration as active ingredients or as excipients as they have been discussed in many studies [4,30,31,32,33]. Semisolid formulations like creams, pastes, gels, and ointments mixed with these clay minerals are used for topical administration. Moreover, they are used as active ingredients with therapeutic action in pharmaceutical products and act as skin (dermatological) protectors [3,33,34], anti-inflammatory [3,33,35], local anesthetics [3,33], antibacterials [3,33], antiseptics [3], disinfectants [3], and for wound healing [3,33]. Therefore, clay minerals increase the ability to protect the skin from the effects of external hazards, harmful agents, toxins, and contaminants [36,37], giving antibacterial and antiseptic action [4] because of their physicochemical properties. On the other hand, their use in cosmetic products for topical administration like sunscreens [3,36,38] and deodorants [3] as active ingredients is achieved with formulations like creams [3,33,39], powders [3,33,39], and emulsions [3]. The main reasons why kaolinite and talc are used in cosmetics as active ingredients are because they produce an opaque effect, are easily absorbed by the skin, have a high adsorption capacity, absorb oils from the skin, are soft, and act as UV filters protecting the skin cells [4,40].

The preparation and quality of pharmaceutical and cosmetic preparations for topical administration with clay minerals of pharmaceutical or cosmetic grade purity is of utmost importance as it is their use as a therapeutic agent for human health and as a cosmetic agent in the care and protection of the body. Their quality must therefore be evaluated, regulated, and complied with by European pharmacopoeia criteria and regulations.

The samples were prepared as semi-solid formulated and specifically as pastes. Nanostuctures such as clay particles, loaded with metals like silver, copper, zinc, and iron are capable of eliminating antibiotic resistant bacteria [41,42,43]. Copper and zinc are the metals chosen to enrich kaolinite and talc due to their antibacterial activity, even in small concentrations as their use in topical applications on the skin has been highlighted in various studies [44,45,46]. Moreover, they are not associated with cytotoxicity and their astounding wound healing ability has been proven [47,48,49,50]. Zinc and copper releasing compounds have been tested and showed good antimicrobial ability [51,52,53,54], while also helping promote cell proliferation [4]. Finally, studies have shown that clays enriched specifically with copper has effective antimicrobial [55,56,57] or antibacterial properties [58]. 

The main purpose of this work was to evaluate the antimicrobial protection via a preservation (preservative) efficacy test of these two clay minerals (kaolinite and talc) of pharmaceutical purity before and after a novel method of metal enrichment by using copper and zinc. Additionally, a significant goal of this study was to evaluate the impact of the metal enriched clay minerals on the resistance of microbial growth when formulated in final products according to the regulations of the European Pharmacopoeia [59] and to what extent they may substitute the use of preservatives. The created formulations (samples) were challenged by exposure to specified types of bacteria, molds, and yeasts to determine whether they were sufficiently preserved.

## 2. Material and Methods

### 2.1. Materials

Pharmaceutical grade clay minerals, kaolin and talc, were purchased from CHEMCO by Syndesmos S.A. (Athens, Greece). In addition, copper chloride (CuCl_2_) and zinc chloride (ZnCl_2_) were purchased from Chem-Lab (Zedelgem, Belgium).

### 2.2. Sample Preparation

#### 2.2.1. Metal Enrichment of Clays

Saturated solutions were prepared by mixing the molar mass of each chloride with 1 L of distilled water.

The chemical modification was achieved by submerging the clay minerals (kaolinite, talc) into the saturated solutions. Thus, kaolinite and talc were mixed with CuCl_2_ and ZnCl_2_ resulting in Cu–kaolinite (CuK), Zn–kaolinite (ZnK), Cu–talc (CuT), and Zn–talc (ZnT). The unmodified kaolinite and talc samples were labeled as K and T, respectively. Specifically, each sample was stirred for 10 min using a magnetic stirrer and then the mixture was centrifuged at 2800 rpm for 10 min. The separated chloride liquid was filtered off and then replaced by fresh chloride solution. Stirring, centrifugation, and refilling were repeated five times [60]. The procedure was repeated several times in order for all or at least almost all the exchangeable cations to be replaced by Cu and Zn cations. One time could probably be enough, but in order to achieve a better result, we repeated the procedure. After that, one last wash took place using distilled water in order to achieve the removal of excess chlorine. Finally, the samples were placed in the oven to dry at 50 °C for two days and then pulverized using an agate mortar and pestle.

#### 2.2.2. Preparation of Pastes

The pastes were created by mixing the clay powders (25% *w*/*w*) with glycerin (5% *w*/*w*), starch (25% *w*/*w*) and deionized water (45% *w*/*w*) and stirred until homogeneity was achieved. The samples could be divided in three groups. The first group contained the pastes with unmodified kaolinite (Kp) and talc (Tp) and served as a control group. The second group contained the modified-clay pastes (CuKp, ZnKp, CuTp, ZnTp), and the third group refers to pastes containing non-modified kaolinite (PHKp) and talc (PHTp) with added phenoxyethanol (1% *w*/*w*, replacing 1% *w*/*w* of water) as the preservative. Each final paste weighed 120 g.

### 2.3. Characterization

#### 2.3.1. X-ray Diffraction Analysis

The mineralogical composition of the samples was determined via x-ray diffraction using a Bruker D8 advance diffractometer (Bruker, Billerica, MA, USA) with nickel-filtered copper Kα radiation and DIFFRAC plus EVA12^®^ software (Bruker-AXS) [61] based on the ICDD (International Centre for Diffraction Data) Powder Diffraction File of PDF-2 2006. The XRD operated at a 2θ range at 2–60° and scanning rate of 2°/min. All samples were prepared using the dropper method.

#### 2.3.2. Scanning Electron Microscope-Energy Dispersive Spectroscopy

Morphological and microelemental analysis of the clay particles were conducted using a scanning electron microscope SEM JEOL 6300 (JEOL, Tokyo, Japan) operating at 30 kV with an energy dispersive spectrometer (EDS). The samples were coated with carbon under vacuum for greater efficiency. Pseudocolorization was used to represent the dispersion of the metals onto the clay surfaces.

#### 2.3.3. X-ray Photoelectron Spectroscopy

The photoemission experiments were carried out in an ultra-high vacuum system (UHV) with a base pressure 1 × 10^−9^ mbar using unmonochromatized MgKα (Berlin, Germany) line at 1253.6 eV. The XPS core level spectra were analyzed using a fitting routine, which can decompose each spectrum into individual mixed Gaussian-Lorentzian peaks after a Shirley background subtraction. The samples were in powder form and pressed in stainless steel pellets.

#### 2.3.4. X-ray Fluorescence

Elemental analysis was performed by x-ray fluorescence (XRF) diffusion using a ZSX PRIMUS II from Rigaku (Tokyo, Japan), with elemental range of Be to U.

### 2.4. Evaluation of Antimicrobial Protection

The evaluation of antimicrobial protection was performed with the preservation (preservative) efficacy test (PET). PET is a reference method, primarily designed for multi-use water-soluble or water-miscible products. It involves, for each micro-organism(s) under test, placing the formulation in contact with a calibrated inoculum, and measuring the changes in the micro-organisms’ population at predefined intervals for a set period and at set temperature. Final results are expressed in log reduction values.

PET for the samples under test, were performed under the mixed protocol. The mixed protocol is based on compendia methods (European and U.S. Pharmacopoeia methods) and consists of challenging the preparation with a mixture of several standard strains, divided in categories of bacteria, yeasts, and molds (Table 1). Unlike the Compendial Pharmacopoeia tests, in which five specified micro-organisms are used as single strain inoculations, the PET mixed protocol includes 11 specified micro-organisms used as grouped (mixed) inocula. The selection of the 11 stains is based on spoiling micro-organisms usually found on cosmetics production sites. Furthermore, applying multi-strain contamination better simulates real-life conditions and further challenges the formulation under test.

The mixed protocol involves, for each group of test micro-organisms, placing the formulation in contact with calibrated inoculums (10 g of the test formulation, 0.1 mL of calibrated inoculums) and measuring the number of surviving micro-organisms at defined intervals (2nd, 7th, 14th, and 28th day) during a period of 28 days.

The containers of inoculation formulation were stored in a dark place at (22.5 ± 2.5 °C). The enumeration method used was the plate count method—pour plate technique, using 1 mL as the inoculation quantity. For each time and each strain, the log reduction value was calculated and compared to the minimum values required for evaluation according to criteria A or B (European Pharmacopoeia, current edition).

The preservative properties of the preparation are adequate if, in the conditions of the test, there is a significant fall or no increase, as indicated by the European Pharmacopoeia criteria. The criteria for the evaluation of PET are given in Table 2 in terms of the log reduction in the number of viable micro-organisms (colony forming units, cfu) against the value obtained for the inoculums. Criteria A express the recommended efficacy to be achieved. In justified cases where Criteria A cannot be attained, for example, for reasons of an increased risk of adverse reactions, Criteria B must be satisfied.

Acceptance criteria for non-sterile pharmaceutical products based upon the total aerobic microbial count (TAMC) and the total combined yeasts/moulds count (TYMC) are given in Table 3.

Prior to each PET, an initial microbial examination test for each product was performed according to the European Pharmacopoeia. More specifically, 10 g of product under test was aseptically transferred to a sterile container and diluted with 90 mL of proper neutralizer (LPT Dilution Broth + 3% Polysorbate 80—Biolife Italiana, Monza, Italy). This is a wide spectrum neutralizer and has been preferred over the Buffered Sodium Chloride Peptone Solution proposed by Eur. Pharmacopoeia, in order to sufficiently suppress the preservation system of the formulations.

After a 15–30 min neutralization step, the plate count method—pour plate technique was used, with 1 mL as the inoculation quantity. For enumeration of total aerobic microbial count (TAMC) and total yeasts-molds count (TYMC), tryptic soy agar and Sabouraud dextrose agar media were used, respectively. The plates were inoculated at 30–35 °C for 3–5 days for TAMC and at 20–25 °C for 5–7 days for TYMC.

## 3. Results and Discussion

### 3.1. X-ray Diffraction Analysis

**Figure 1:**
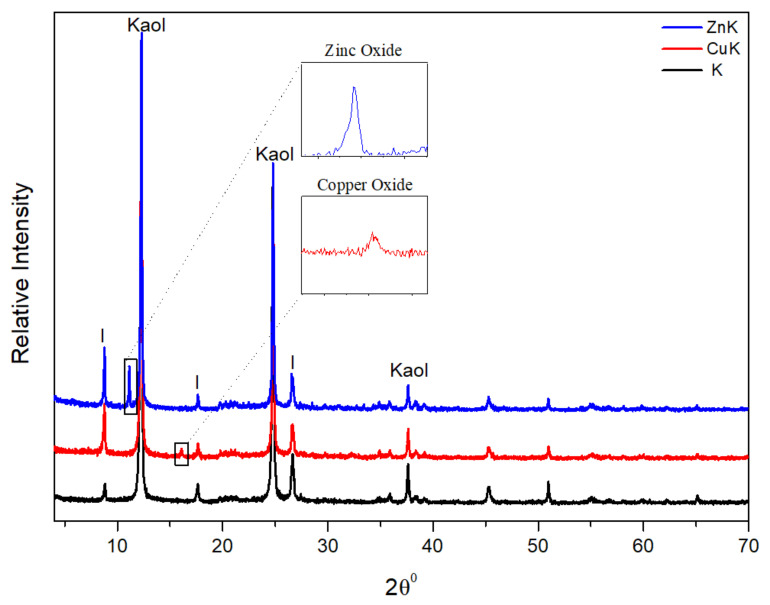
X-ray diagrams of unmodified kaolinite (K), copper modified kaolinite (CuK), and zinc modified kaolinite (ZnK) (Legend: Kaol.: Kaolinite, I: Illite).

**Figure 2:**
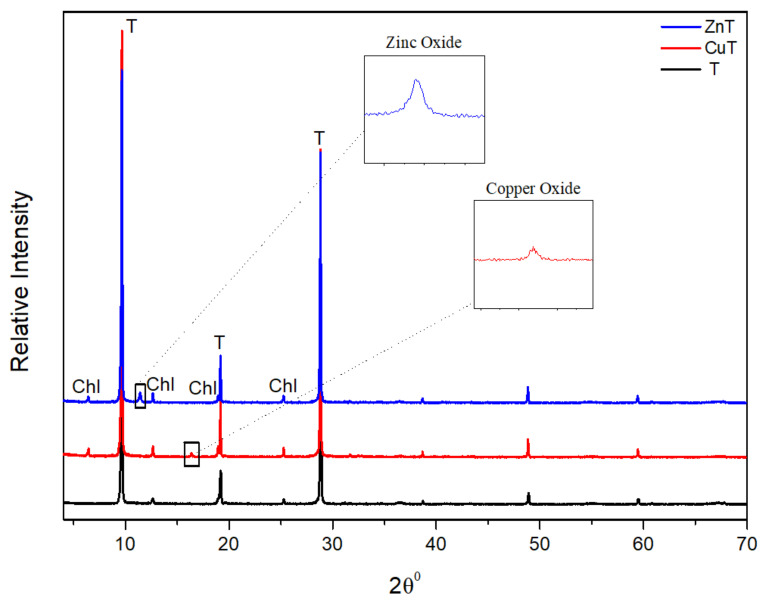
X-ray diagrams of unmodified talc (T), copper modified talc (CuT), and zinc modified talc (ZnT) (Legend: Chl: Chlorite).

The x-ray diffraction patterns show that both the kaolin (Figure 1) and talc (Figure 2) samples had the expected phases in a pharmaceutical grade clay. Therefore, in K, CuK and ZnK peaks at 7.19 Å revealed the dominant phase of kaolinite and small amounts of illite at 9.86 Å. No changes in the diffraction patterns and d_(001)_ spacing between the unmodified and modified kaolinite imply that the modification did not alter the internal structure of the crystals [62]. This could further imply that the adsorption of copper and zinc ions occurred on kaolinite surfaces instead of intercalated between the sheets [63].

As for T, CuT and ZnT talc could be found at 9.17 Å, while traces of chlorite were detected at 13.95 Å. Similarly, no changes in the diffraction patterns imply no structural alteration of the crystals. Comparing the modified samples to their unmodified versions, some new peaks were revealed in CuK (16.15°), ZnK (11.15°), CuT (16.23°), and ZnT (11.33°). This implies the formation of new phases during the chemical modification. Specifically, relatively low peaks indicating small amounts of salt compounds, probably oxides. No significant differences were found between examined FTIR spectra of modified and non-modified clay minerals (Figures S1 and S2).

### 3.2. Scanning Electron Microscope (SEM)-Energy Dispersive Spectroscopy

**Figure 3:**
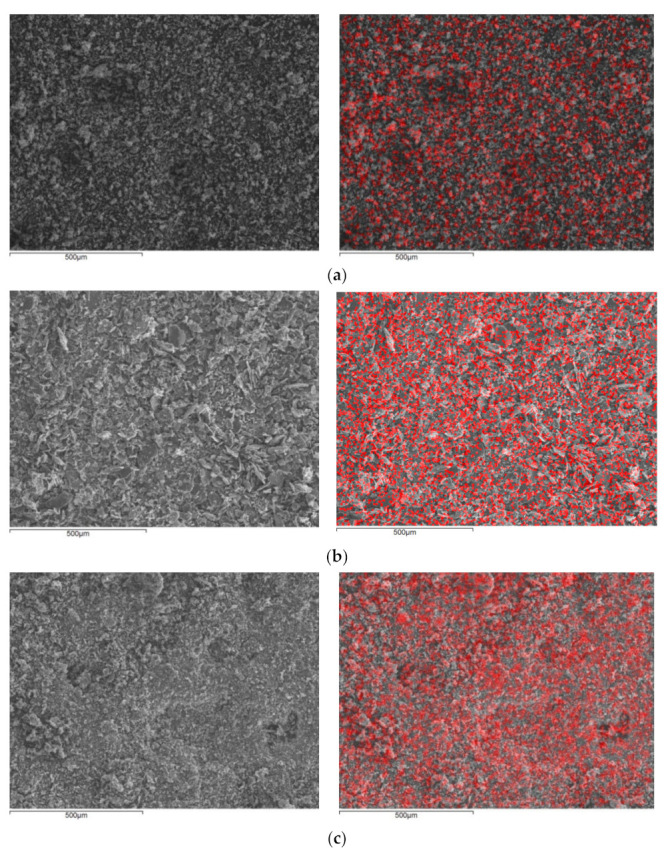

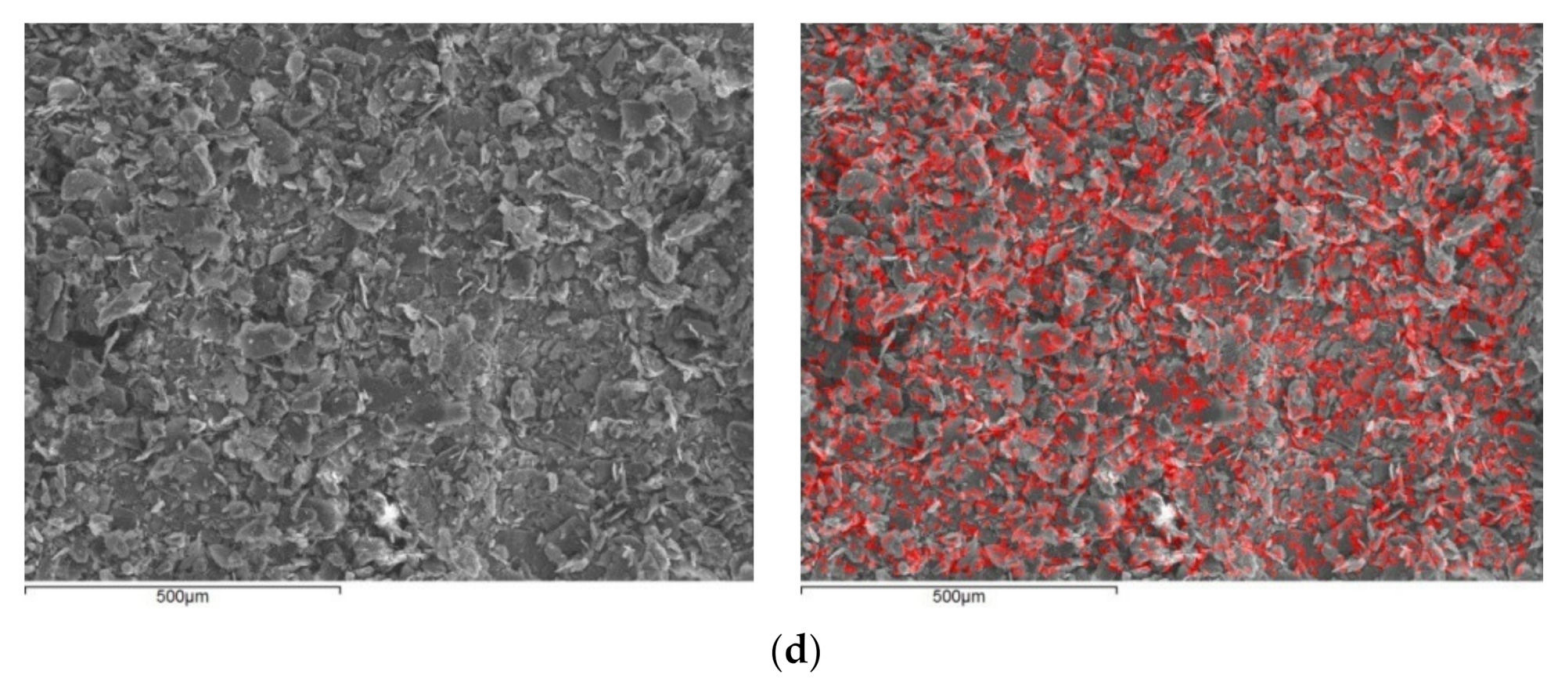
Images from scanning electron microscopy (SEM) showing before (left) and after (right) elemental mapping (**a**), CuK (**b**), CuT (**c**), ZnK, and (**d**), ZnT. The distribution of copper and zinc, respectively, is indicated by red dots.

Through SEM images, the morphology and size of the crystals were observed (Figure 3). Cu–Kaolinite and Zn–Kaolinite particles are aggregates of booklet-like stacked platelets, while their size ranges from 250 nm to 15 μm. Talc’s particles mostly resemble huddled clustered stacks, while some flat tabular crystals are also present. The particles’ sizes range from 500 nm to 100 μm according to SEM observation.

Elemental mapping revealed the distribution of zinc and copper on the clay particles. Pseudocolors are used to represent the presence of the metals. As desired, in all the samples, the distribution seems to be both homogenous and dense. In all cases, no large aggregates of either metal were observed. It should be noted that neither illite nor chlorite were detected in any of the images.

### 3.3. X-ray Fluorescence Spectroscopy Analysis 

XRF results show the dominance of Si and Al in the kaolin samples and Si and Mg in the talc samples, which is to be expected as kaolinite Al_2_Si_2_O_5_(OH)_4_ and talc Mg_3_Si_4_O_10_(OH)_2_ are the main phases of said samples, respectively. Elevated potassium in kaolin samples could be due to the presence of illite, while elevated iron values in talc samples could be attributed to chlorite (Table 4).

Trace element composition revealed that the modified clays were successfully enriched with copper and zinc. Therefore, CuK and CuT showed an abundance of copper, while ZnK and ZnT showed an abundance of zinc. It should be noted that in Cu-treated samples, the amounts of other metals like Sc and Ba were higher, which indicates that they were present in the salt solution and therefore adsorbed by the clays via the cation exchange process (Table 5).

### 3.4. X-ray Photoelectron Spectroscopy

**Figure 4:**
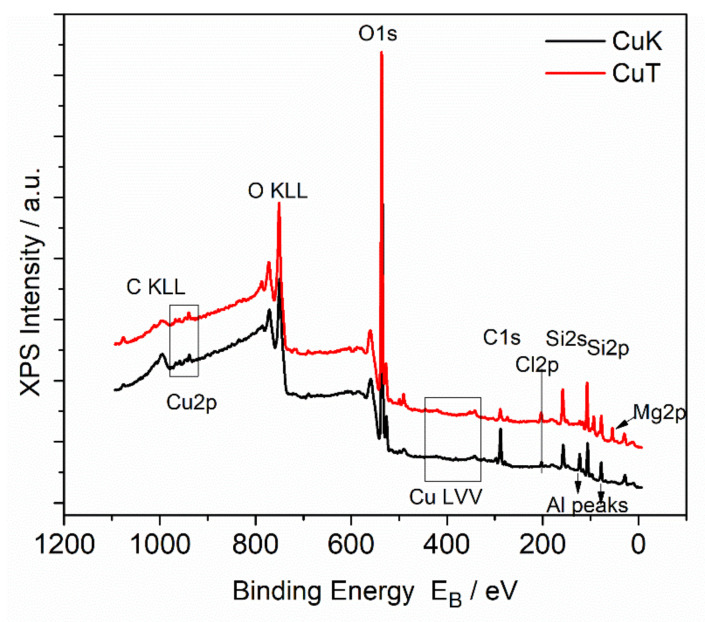
X-ray photoelectron spectroscopy (XPS) survey scans of the CuK and CuT samples.

**Figure 5:**
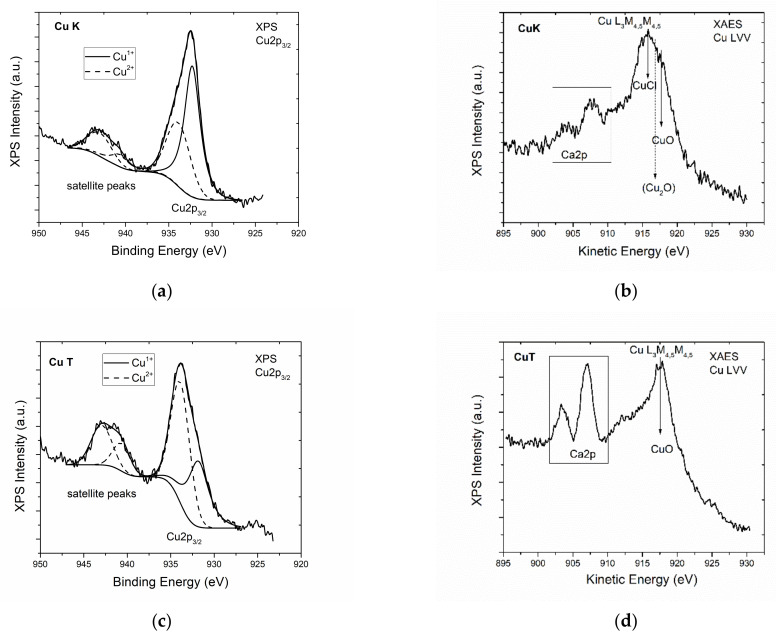
Deconvoluted high resolution Cu2p spectrum from the (**a**,**b**) CuK and (**c**,**d**) CuT samples.

**Figure 6:**
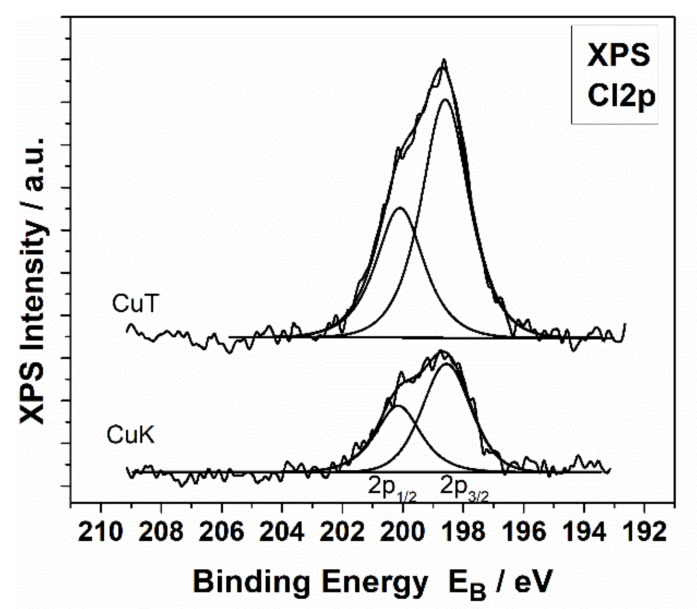
High resolution Cl2p spectrum from the CuK and CuT samples.

Figure 4 shows the survey scans of the CuK and CuT samples where Cu, O, Cl, C, and Si peaks are presented in all samples. Additionally, Mg peaks on the CuT sample surface whereas Al peaks on CuK surfaces were present. Figure 5 shows the deconvoluted spectra of the Cu 2p photoelectron peak and the CuLVV Auger peak of the CuK and CuT samples. The Cu2p3/2 peak is deconvoluted into two components assigned to Cu^1+^ (CuClO_4_ or CuCl) and to Cu^2+^(CuO). It must be mentioned that the binding energy of CuO and CuClO_4_ were identical, thus the absence of CuO could not be excluded. In contrast, the Auger kinetic energy differed, thus the modified Auger parameter was different. Nevertheless, the CuL_3_M_4.5_M_4.5_ Auger peak was difficult to analyze, but taking into account the presence of Cl in all samples and the kinetic energy of CuL_3_M_4.5_M_4.5_, we came to the conclusion that CuCl or CuClO_4_, which were identified by XRD dominates on CuK samples [64]. Figure 6 shows the Cl2p peak of the CuK and CuT samples. The binding energy of Cl2p3/2 at 198.5eV was assigned to the Cu chloride (CuCl) [65]. There was no significant CuClOx on the surface that would give rise to Cl2p3/2 peaks at energies approximately 206–208 eV [66]. We concluded that on the surface, only CuCl was present. Moreover, a peak of Mg2p was present, which indicates the presence of Mg atoms. A rough estimation of the % chemical states concentration of the CuK and CuT samples was performed based on internal reference and the results are shown in Table 6.

**Figure 7:**
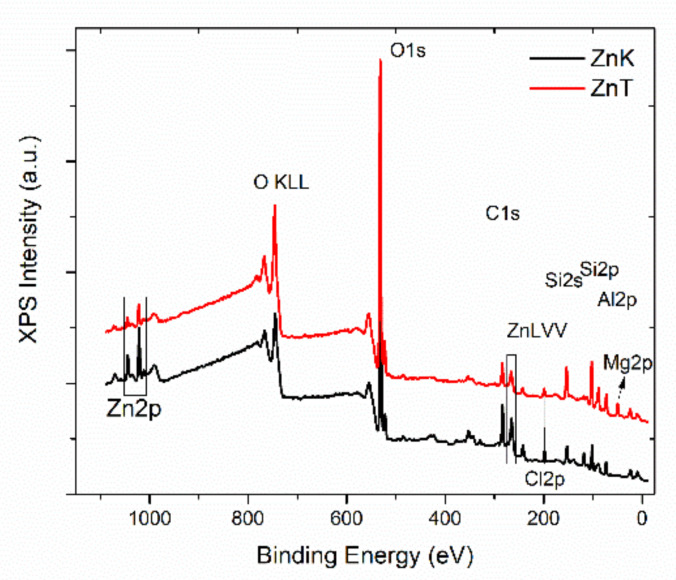
XPS survey scans of the ZnK and ZnT samples.

Figure 7 shows the survey scans of the ZnK and ZnT samples where Zn, O, Cl, C, and Si peaks are presented in all samples. Additionally, Mg peaks were present on the ZnT sample surface whereas Al peaks on the ZnK surfaces are present.

**Figure 8:**
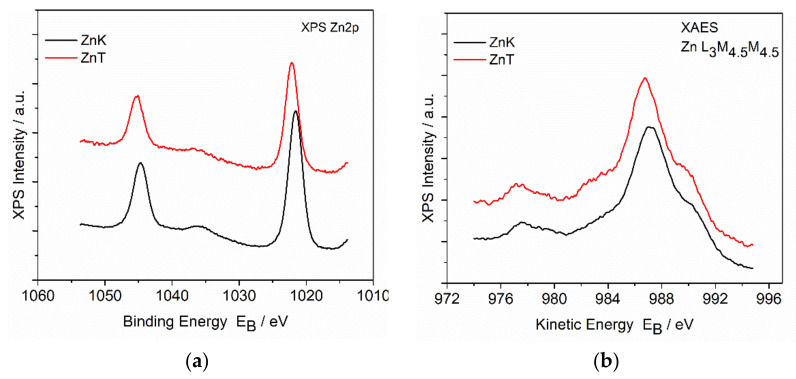
High resolution (**a**) Zn2p and (**b**) ZnL3M4.5M4.5 spectra from of the ZnK and ZnT samples.

Figure 8 shows the detailed spectra of the Zn2p photoelectron peak and ZnLMM Auger peak. It is known that the Zn2p3/2 spectrum of zinc oxide overlaps with the metal peak binding energy. For this reason, the chemical state determination can be made by using the modified Auger parameter (defined by the sum of the Binding energy of Zn2p3/2 and the kinetic energy of ZnL_3_M_4.5_M_4.5_). However, due to the spectral overlap, mixed systems of different oxide compounds is very difficult to quantify. In our samples, the modified Auger parameter of ZnK was 2009.8 eV and that of ZnT was 2009.9 eV, assigned to zinc oxide (ZnO) [67] and zinc perchlorate (Zn(ClO_4_)_2_) [68].

It should be noted that the XPS survey scans measure binding energy from which it is not possible to define a phase. Theoretically, the binding energy of the suggested CuO or ZnO could in fact represent the bond between adsorbed copper (Cu^2+^) or zinc (Zn^2+^) ions and silicon tetrahedra’s oxygens. Additionally, this theory is supported by the absence of CuO or ZnO in x-ray diagrams as no crystalline form was in adequate amounts to be detected. In the case of Cu^1+^, the corresponding compound CuClO_4_ was identified in the x-ray diagrams, meaning that the copper salt is present in the copper modified samples. Similarly, Zn(ClO_4_)_2_ was detected in both zinc modified samples.

### 3.5. Challenge Test

The microbial load of all samples was evaluated before the challenge test, as this needs to be as low as possible to conduct trustworthy results. The unmodified clay pastes (Kp and Tp) have developed a total aerobic microbial count >3 × 10^3^ cfu/g five days after their preparation (Table 7). Jou et al. (2016) [63] and Holesova et al. (2013) [69] also reported that natural clay minerals (e.g. kaolinite), showed no antibacterial effect. According to the literature, clay minerals are loaded with inorganic species exhibiting antibacterial properties such as copper ions [70,71] and zinc ions [72,73]. However, this observation was not observed for the CuTp and ZnKp pastes that were also found with a microbial load of total anaerobic microbial count >3 × 10^3^ cfu/g as well as molds and yeasts 4 × 10 and >3 × 10^2^, respectively, five days after their preparation (Table 7).

These results render the four paste samples to not qualify for the challenge test as they surpass the limits (Table 3) for cutaneous uses given by the European Pharmacopoeia 10.0.

In addition, the ZnTp paste presented predominant strains (*St. epidermis, K. rhizophila*). Regarding CuKp and ZnTp pastes, better antibacterial properties have been exhibited. In Table 8, the microbial count is presented starting from the initial inoculation and continuing with the 2nd, 7th, 14th, and 28th day to track the growth of each microorganism for the pastes CuKp and ZnTp as well as for the clay minerals loaded with the organic antibacterial agent (phenoxythanol). It is well known that although the natural clay minerals show no antibacterial effect, they could kill or adsorb bacteria when some antibacterial material is intercalated in or adsorbed on them [69,74]. Specifically, the samples containing phenoxyethanol (PHKp, PHTp) passed the test for bacteria, molds, and yeasts, meeting Criteria A for all said organisms (Table 9).

Their preservation capacity of phenoxyethanol was excellent as the bacterial total count of the samples went under 100 cfu/g within two days and was eradicated reaching the seventh day. As for molds and yeasts, the preservation effect was sufficient and the most significant reduction occurred after seven or 14 days, except for the excellent antimicrobial protection of PHTp on yeasts, which mirrored the bacterial reduction.

**Figure 9:**
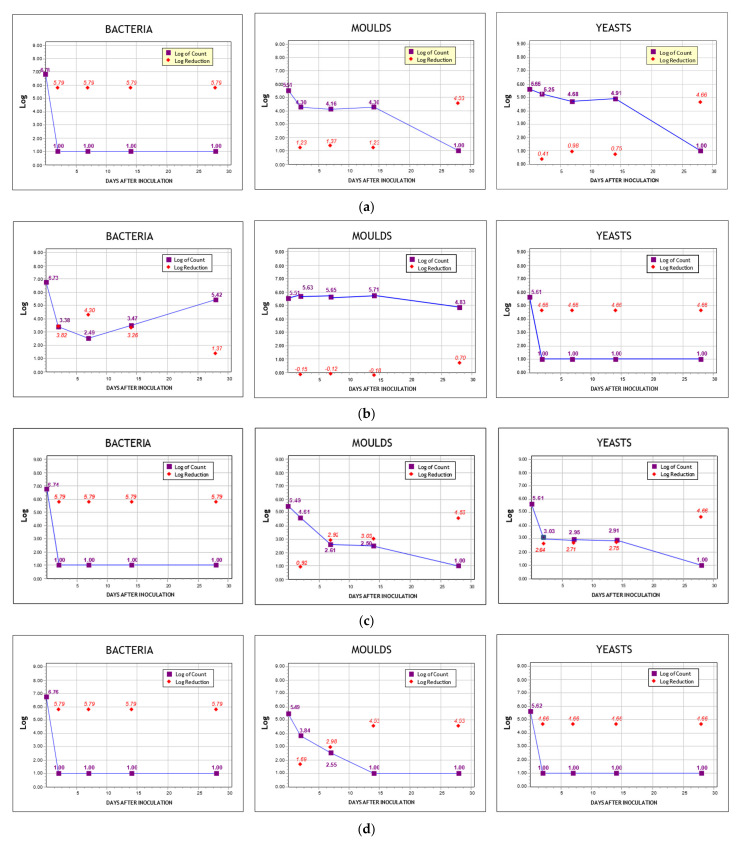
Logarithmic reduction charts of each sample against bacteria, molds, and yeasts. (**a**) CuKp, (**b**) ZnTp, (**c**) PHKp, and (**d**) PHTp.

As for the chemically modified clay samples, none managed to satisfy all criteria at once. Even so, CuKp showed excellent preservation activity against bacteria, reducing all the inoculated bacteria within seven days, while also demonstrating an adequate reduction of cfu for molds capable of satisfying the criteria B of the PhEur. For yeast, no criteria were met. In contrast, ZnTp failed to meet all criteria for bacteria and molds, but it showed significant antimicrobial protection on yeasts, completely reducing the total count in seven days, thus satisfying Criteria A. The logarithmic reduction of cfu and the score for each sample according to the relevant criteria are summarized in Table 8. Total counts and reduction rates are plotted logarithmically in Figure 9.

## 4. Conclusions

In this study, pastes composed by copper or zinc loaded kaolin and talc powders were prepared to evaluate their resistance to microbial growth. The mineralogical purity of the kaolin and talc powders was assessed via x-ray diffraction, while the homogenous dispersion of each metal on the clay surfaces was observed using SEM-EDS. Additionally, the XRF results displayed the abundance of each respective metal in each sample. Finally, the Cu^2+^ adsorbed on clay minerals can be assumed from the XPS results.

Consequently, none of the paste samples utterly met the criteria of acceptance for cutaneous use given by the European Pharmacopoeia. However, the antimicrobial protection associated to most of the samples was noticeable and, in some cases, excellent. Copper loaded kaolin showed remarkable results against bacteria and molds meeting the relevant Criteria A and B, respectively. Although unable to meet any criteria for yeasts, it still performed well enough to credit its antifungal ability. Zinc loaded talc performed excellently against yeasts, with identical results to the phenoxyethanol sample. Thus, materials like these are seemingly promising for the creation of products characterized by lesser usage of preservatives.

## Figures and Tables

**Table 1:** Types of microorganisms tested.

Test Organisms					
Gram-positive bacteria	*St. aureus* ATCC 6538		*St. epidermitis* ATCC 12228	*Kokuria rhizophilia* ATCC 9341	
Gram-negative bacteria	Enterobacteria		Pseudomonas		
	*E. coli* ATCC 8739	*Enterbac. Gergoviae* ATCC 33028	*Pseudom. aeruginosa* ATCC 9027	*Burkhold. cepacian* In house	*Pseudom. luteola* ATCC 43330
Yeasts	*Candida albicans* ATCC 10231	Molds	*Aspergillus brasiliensis* ATCC 16404	*Penicillium aurantiogriseum* ATCC 16025	

ATCC (Wesel, Germany).

**Table 2:** European Pharmacopoeia, Criteria A and B, for cutaneous application, minimum reduction in log units.

Criteria A				
Time	2nd Day	7th Day	14th Day	28th Day
Bacteria	2	3	-	NI
Yeasts	-	-	2	NI
Molds	-	-	2	NI
**Criteria B**				
Time	2nd Day	7th Day	14th Day	28th Day
Bacteria	-	-	3	NI
Yeasts	-	-	1	NI
Molds	-	-	1	NI

NI: No Increase.

**Table 3:** Acceptance criteria for microbiological quality of non-sterile dosage forms (European Pharmacopoeia 10.0).

Route of Administration	Total Aerobic Microbial Count (cfu/g or cfu/mL)	Total Combined Yeasts/Molds Count (cfu/g or cfu/mL)	Specified Microorganism
Cutaneous use	10^2^	10^1^	Absence of Staphylococcus aureus (1 g or 1 mL) Absence of Pseudomonasaeruginosa (1 g or 1 mL)

When an acceptance criterion for microbiological quality is prescribed, it is interpreted as follows: 10^1^ cfu: maximum acceptance count = 20, 10^2^ cfu: maximum acceptance count = 200, 10^3^ cfu: maximum acceptance count = 2000, and so forth.

**Table 4:** Major element composition (% oxides) (XRF) of K, CuK, ZnK, T, CuT, and ZnT powder samples.

Oxide	K	CuK	ZnK	T	CuT	ZnT
SiO_2_	44.58	42.79	45.52	64.71	60.62	56.21
Al_2_O_3_	35.88	33.37	35.6	0.53	0.59	0.57
Fe_2_O_3_	0.82	0.90	0.84	3.89	3.85	3.63
MnO	0.01	0.01	0.01	0.02	0.01	0.02
MgO	0.39	0.38	0.37	27.62	27.41	27.04
CaO	0.28	0.23	0.25	0.38	0.28	0.36
Na_2_O	0.15	0.13	0.47	0.07	0.08	0.29
K_2_O	2.48	2.40	2.51	BDL	0.00	BDL
TiO_2_	0.03	BDL	0.03	BDL	BDL	BDL
P_2_O_5_	0.12	0.10	0.1	BDL	0.01	BDL
LOI	11.32	12.08	11.48	5.09	6.46	6.59
Sum	96.06	92.39	97.18	102.31	99.31	94.71

BDL: Below detection limit. LOI: Loss of ignition.

**Table 5:** Trace element composition (ppm) (XRF) of K, CuK, ZnK, T, CuT, and ZnT powder samples.

Element	K	CuK	ZnK	T	CuT	ZnT
Sc	10	1866	11	8	1638	9
Cr	7	6	8	67	135	77
Co	BDL	BDL	BDL	BDL	BDL	BDL
Cu	BDL	23467	BDL	BDL	20603	BDL
Zn	35	1916	23148	25	1715	47365
Sr	108	121	106	6	15	7
Y	74	93	58	36	48	30
Ba	218	8994	210	31	7717	44
Hf	7	26	6	5	24	6
Pb	BDL	77	BDL	BDL	55	BDL

BDL: Below detection limit.

**Table 6:** % composition of Cu^2+^ and Cu^1+^ (CuCl) of the CuK and CuT samples.

Sample	Cu^2+^	Cu^1+^ (CuCl)
CuK	49	51
CuT	86	14

**Table 7:** Initial microbial load test according to the European Pharmacopoeia method.

Paste	Parameter	Result (cfu/g)	Limits (cfu/g)
Kp	Total Aerobic Microbial Count	>3.0 × 10^3^	<1.0 × 10^2^
	Molds & Yeasts	<10	<10
Tp	Total Aerobic Microbial Count	>3.0 × 10^3^	<1.0 × 10^2^
	Molds & Yeasts	<10	<10
CuTp	Total Aerobic Microbial Count	>3.0 × 10^3^	<1.0 × 10^2^
	Molds & Yeasts	4.0 × 10^1^	<10
ZnKp	Total Aerobic Microbial Count	>3.0 × 10^3^	<1.0 × 10^2^
	Molds & Yeasts	>3.0 × 10^2^	<10

**Table 8:** Mixed culture–preservation efficacy test.

Paste	Parameter	Sterility Control	Inoculation	0 Time	2nd Day	7th Day	14th Day	28th Day
CuKp	Bacteria	<10	6.2 × 10^6^	6.1 × 10^6^	<100	<10	<10	<10
	Molds	<10	3.4 × 10^5^	3.3 × 10^5^	2.0 × 10^4^	1.5 × 10^4^	2.0 × 10^4^	<10
	Yeasts	<10	4.6 × 10^5^	4.5 × 10^5^	1.8 × 10^5^	4.8 × 10^5^	8.2 × 10^5^	<10
ZnTp	Bacteria	<10	6.2 × 10^6^	5.4 × 10^6^	2.4 × 10^3^	3.1 × 10^2^	3.0 × 10^3^	2.6 × 10^5^
	Molds	<10	3.4 × 10^5^	3.3 × 10^5^	4.8 × 10^5^	4.5 × 10^5^	5.2 × 10^5^	6.8 × 10^4^
	Yeasts	<10	4.6 × 10^5^	4.1 × 10^5^	<100	<10	<10	<10
PHKp	Bacteria	<10	6.2 × 10^6^	5.5 × 10^6^	<100	<10	<10	<10
	Molds	<10	3.4 × 10^5^	3.1 × 10^5^	4.1 × 10^4^	4.1 × 10^2^	3.2 × 10^2^	<10
	Yeasts	<10	4.6 × 10^5^	4.1 × 10^5^	1.1 × 10^3^	9.0 × 10^2^	8.2 × 10^2^	<10
PHTp	Bacteria	<10	6.2 × 10^6^	5.7 × 10^6^	<100	<10	<10	<10
	Molds	<10	3.4 × 10^5^	3.1 × 10^5^	6.9 × 10^3^	3.6 × 10^2^	<10	<10
	Yeasts	<10	4.6 × 10^5^	4.2 × 10^5^	<100	<10	<10	<10

**Table 9:** Criteria of acceptance (evaluation criteria).

Paste	Log Reduction	2nd Day	7th Day	14th Day	28th Day	Criterion Α	Criterion B	Test Result
CuKp	Bacteria	6.79	6.79	6.79	6.79	✓		Failed, does not meet the relevant A/B criteria
	Molds	1.23	1.37	1.23	4.63		✓	
	Yeasts	0.41	0.95	0.75	4.66			
ZnTp	Bacteria	3.82	4.30	1.92	1.37			Failed, does not meet the relevant A/B criteria
	Molds	−0.15	−0.12	−0.18	0.70			
	Yeasts	4.06	4.06	4.06	4.06	✓		
PHKp	Bacteria	5.79	5.79	5.79	5.79	✓		Satisfactory, meet the relevant A criteria
	Molds	0.92	2.92	3.03	3.53	✓		
	Yeasts	2.64	2.71	2.75	4.66	✓		
PHTp	Bacteria	5.79	5.79	5.79	5.79	✓		Satisfactory, meet the relevant A criteria
	Molds	1.69	2.06	4.55	4.55	✓		
	Yeasts	4.06	4.06	4.06	4.06	✓		

## Data Availability

Data sharing is not applicable to this article.

## Supplementary Materials

The following are available online at https://www.mdpi.com/1996-1944/14/5/1173/s1, Figure S1: FTIR spectra diagram of K, CuK, and ZnK powder samples, Figure S2: FTIR spectra diagram of T, CuT, and ZnT powder samples.
