# Supplementary material for: Evaluation of the Antimicrobial Protection of Pharmaceutical Kaolin and Talc Modified with Copper and Zinc
Source: Materials (Basel). 2021 Mar 2;14(5):1173. doi: 10.3390/ma14051173 (PMC7958952; doi:10.3390/ma14051173)
Supplement: Supplementary file 1 [file materials-14-01173-s001.pdf]

materials

Supplementary Materials

Evaluation of the Antimicrobial Protection of Pharmaceutical Kaolin and Talc Modified with Copper and Zinc

Fotini Martsouka [1,*], Konstantinos Papagiannopoulos [1], Sophia Hatziantoniou [2], Martin Barlog [3], Giorgos Lagiopoulos [4], Athanasia G. Tekerlekopoulou [5] and Dimitrios Papoulis [1]

Citation: Martsouka, F.; Papagiannopoulos, K.; Hatziantoniou, S.; Barlog, M.; Lagiopoulos, G.; Tekerlekopoulou, A.G.; Papoulis, D. Evaluation of the Antimicrobial Protection of Pharmaceutical Kaolin and Talc Modified with Copper and Zinc . *Materials* **2021**, *14*, 1173. https://doi.org/10.3390/ma14051173

Academic Editor: Rosaria Anna Picca

Received: 15 January 2021
Accepted: 25 February 2021
Published: 2 March 2021

Publisher's Note: MDPI stays neutral with regard to jurisdictional claims in published maps and institutional affiliations.

Copyright: © 2021 by the authors. Licensee MDPI, Basel, Switzerland. This article is an open access article distributed under the terms and conditions of the Creative Commons Attribution (CC BY) license (http://creativecommons.org/licenses/by/4.0/).

1. Material and Methods

1.1. Characterization

1.1.1. Fourier Transform Infrared Spectroscopy

The infrared spectra were obtained on a Nicolet 6700 Fourier Transform Infrared (FTIR) spectrometer from Thermo Scientific™. IR (Infrared spectroscopy) source (wolfram wire), KBr beam splitter, and DTGS (Detector for Fourier Transform Spectroscopy) detector was used for measurement in the middle IR region (MIR 4000–400 cm^{-1}). MIR (mid-infrared spectroscopy) transmission spectra were collected using the KBr pellet press technique (0.5–1 mg of sample homogenized with 200 mg KBr). The pellets were heated overnight at 140 °C to minimize the content of adsorbed water. For each sample, 128 scans with a resolution of 4 cm^{-1} were recorded.

2. Results and Discussion

2.1. Fourier Transform Infrared Spectroscopy

The FTIR spectra of both unmodified and modified versions of kaolin and talc were studied as a supplementary method to XRD. The samples of kaolin revealed four well resolved kaolinite absorption bands in the OH stretching region of 3696–3619 cm^{-1} and two OH-bending bands at 914 cm^{-1} corresponded to inner hydroxyl groups and at 938 cm^{-1} to vibrations of surface OH groups (Figure S1). The three higher frequency bands were assigned to the outer hydroxyls and band at ~3619 cm^{-1} was attributed to the vibration of inner surface hydroxyls [1]. The region between 1032–1008 cm^{-1} was assigned to antisymmetric in plane Si–O–Si stretching vibrations, the band at 1113 cm^{-1} related to in-phase stretching of apical Si–O bonds of the kaolinite, while the perpendicular Si–O bending mode was attributed to 793, 755, and ~696 cm^{-1} vibration bands [2,3]. The absorption bands due to Si–O–Al, Si–O–Si, and Si–O deformations were present at 538, 469, and 429 cm^{-1} [3].

In the spectra of talc samples (Figure S2) the sharp bands at 3676 cm^{-1} and at 669 cm^{-1} corresponded to stretching and bending of Mg_3OH units overlapped with a weak shoulder at ~690 cm^{-1} assigned to Si–O perpendicular vibrations. Two broad weak bands located at ~3338 cm^{-1} and ~3446 cm^{-1} were not associated with the talc nor the kaolinite structure, but came from vibrational modes of weakly bound water on the surface [2,4]. The spectrum of talc showed a very weak band at 3660 cm^{-1} due to the presence of iron, which relates to the $Mg_2Fe^{2+}OH$ stretching mode. The Si–O in plane stretching mode occurred at ~1018 cm^{-1} with a very weak shoulder at ~1045 cm^{-1} assigned to the perpendicular Si–O stretching mode, appearing for talc with a very low iron content. The absorption bands at 534 cm^{-1} related to perpendicular Mg–O vibration, at 466 cm^{-1} to translation vibration of the OH groups, and at 450 cm^{-1} to the Si–O–Si bending vibration, respectively [2]. No significant differences were found between the examined FTIR spectra of modified and non-modified clay minerals.

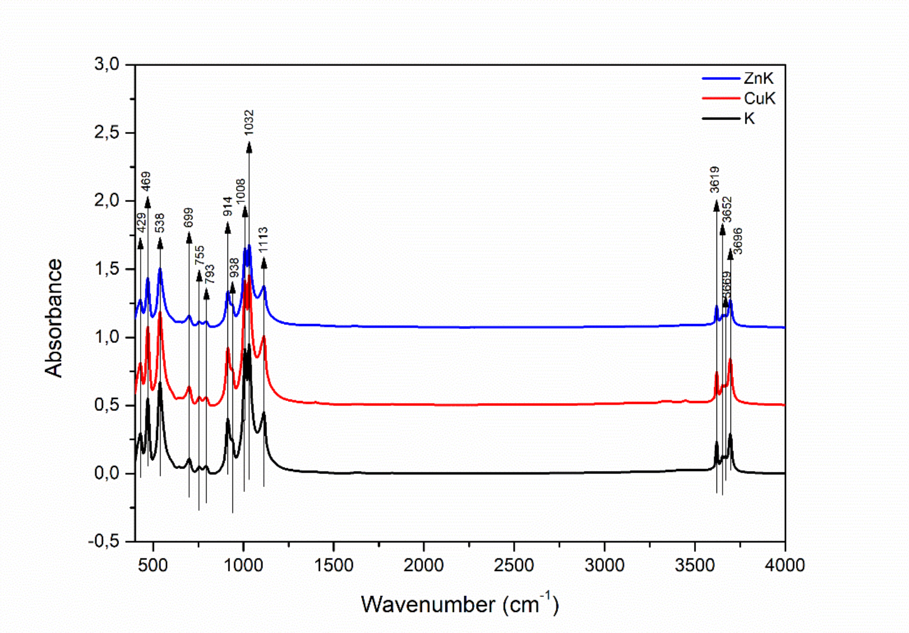

Figure S1. Fourier transform infrared (FTIR) spectra diagram of the K, CuK, and ZnK powder samples.

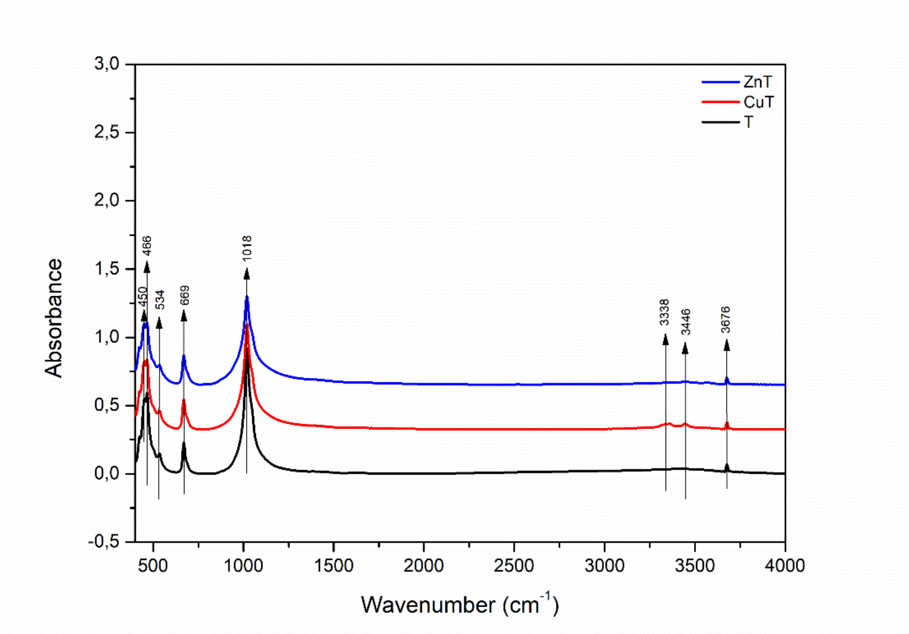

Figure S2. FTIR spectra diagram of T, CuT, and ZnT powder samples.

3. Conclusions

The FTIR spectra diagrams indicated no substantial structural change after the enrichment process.

References

1. Madejová, J.; Kečkéš, J.; Pálková, H.; Komadel, P. Identification of components in smectite/kaolinite mixtures. *Clay Miner.* **2002**, *37*, 377–388.
2. Madejová, J.; Gates, W.; Petit, S. IR Spectra of Clay Minerals. *Dev. Clay Sci.* **2017**, *8*, 107–149, doi:10.1016/B978-0-08-100355-8.00005-9.

3. Vaculíková, L.; Plevová, E.; Vallová, S.; Koutnik, I. Characterization and differentiation of kaolinites from selected Czech deposits using infrared spectroscopy and differential thermal analysis. *Acta Geodyn. Geomater.* **2011**, *8*, 59–67.
4. Blanchard, M.; Méheut, M.; Delon, L.; Poirier, M.; Micoud, P.; Le Roux, C.; Martin, F. Infrared spectroscopic study of the synthetic Mg–Ni talc series. *Phys. Chem. Miner.* **2018**, *45*, 843–854, doi:10.1007/s00269-018-0966-x.
